# Putting Fungi to Work: Harvesting a Cornucopia of Drugs, Toxins, and Antibiotics

**DOI:** 10.1371/journal.ppat.1003950

**Published:** 2014-03-13

**Authors:** Ulrich Kück, Sandra Bloemendal, Ines Teichert

**Affiliations:** 1 Christian Doppler Laboratory for Fungal Biotechnology, Ruhr-Universität Bochum, Bochum, Germany; 2 Lehrstuhl für Allgemeine und Molekulare Botanik, Ruhr-Universität Bochum, Bochum, Germany; Duke University Medical Center, United States of America

Fungi belong amongst the severest pathogens of humans, animals, and plants. For example, *Candida* spp. and *Aspergillus* spp. account for most invasive mycoses, and such infections are associated with high rates of mortality in hematology and oncology patients. Worrisomely, it has been estimated that 4% of all patients who die in hospitals die of invasive aspergillosis and 2% die of candidiasis [1]. Moreover, in agriculture, filamentous fungi are mainly responsible for severe loss of crops worldwide, destroying over 125 million tons of rice, wheat, maize, potatoes, and soybeans each year. Calculations for 2011 predicted that prevention of these losses would be sufficient to feed 600 million people [2]. However, due to the common negative perception of fungi as pathogens, we often lose sight of the beneficial role of fungi as producers of a cornucopia of life-saving drugs.

## Janus-Like Role of Fungi

Some fungi are at the same time a “friend” and “foe” of humans. A good example of a filamentous fungus with a Janus-like role is *A. terreus*, which has opposing effects on human health. As a pathogen, this fungus is the third most significant cause of invasive aspergillosis, having a reported frequency of 3–12.5%. In addition, it also produces several mycotoxins that can be the cause of food spoilage, especially of cereals and nuts in tropical and subtropical climates. However, this fungus can also produce a number of organic acids and secondary metabolites that, due to their clinical relevance, are of major biotechnological and pharmaceutical interest. Lovastatin, a polyketide derivative, is the most important one because of its cholesterol-lowering properties (Table 1) and has been used successfully for decades to treat coronary artery disease, a prevalent cause of premature death in the Western world [3]. Moreover, this fungus can also produce gliotoxin, which acts as an immunosuppressive drug (Table 1).

**Table 1:** Selected fungal products from different compound classes, their application, and producers.

Product (Compound Class)	Application	Organism
Cephalosporin (nonribosomal peptide derivative)	Antibiotic	*Ac. chrysogenum*
Lovastatin (polyketide)	Cholesterol-lowering drug	*A. terreus*
Mycophenolic acid (meroterpenoid)	Immunosuppressive	*P. brevicompactum*
Penicillin (nonribosomal peptide derivative)	Antibiotic	*P. chrysogenum*
Compactin (polyketide)	Cholesterol-lowering drug	*P. citrinum*
Cyclosporin A (nonribosomal peptide derivative)	Immunosuppressive	*Tolypocladium inflatum*
Gliotoxin (nonribosomal peptide derivative)	Immunosuppressive	*A. terreus*

Two other representative examples of fungi with a Janus-like character are serious plant pathogens. *Claviceps purpurea* is the producer of ergot alkaloids, naturally occurring mycotoxins that contaminate grains. Consumption of contaminated food causes ergotism, a disease that resulted in more than 40,000 deaths in Europe in the Middle Ages. The toxic and therapeutic effects of ergot alkaloids have been known for centuries, and, nowadays, alkaloids form the basis of many synthetic drugs that treat the various symptoms of migraines [4]. A second example is *Ashbya gossypii*, a major plant pathogen that causes stigmatomycosis of cotton and citrus fruits in tropical and subtropical countries. In the 1940s, *Ash. gossypii* was found to be a high-level producer of riboflavin, also called vitamin B_2_. This essential vitamin cannot be synthesized by humans and must be supplied by food and dietary supplements. Nowadays, the industry uses *Ash. gossypii* as a producer of riboflavin [5], since microbial fermentation with a yearly production yield of about 9,000 tons is economically more feasible than chemical synthesis.

## The Treasure Trove: Secondary Metabolites Produced by Filamentous Fungi

From the numerous above-mentioned examples, it is clear that filamentous fungi have an extensive metabolism and produce a wealth of bioactive compounds. Many of their secondary metabolites contribute substantially to the pathogenicity of fungi and to the toxicity of contaminated food and crops [2], [6]. Notably, secondary metabolites differ from primary metabolites in that they (1) are not directly derived from any intermediary metabolism, (2) are often produced during a specific morphogenetic program, and (3) are not essential for survival of the producing organisms [7]. However, secondary metabolites may provide abiotic (melanins) and biotic properties as antifungal, antibacterial, and insecticidal agents [7]–[9]. Importantly, the content and composition profile of secondary metabolites differs from species to species and even within strains of a single species.

Secondary metabolites are highly diverse, and their biosynthesis genes are often organized in clusters and controlled by transcriptional regulation and chromatin remodeling [10]. Currently, they can be classified into different compound classes [7], including polyketides, fatty acid derivatives, and nonribosomal peptides that are produced by large modular enzymes, as well as terpenes and alkaloids. Moreover, several hybrid secondary metabolites are also known, namely meroterpenoids, which consist of polyketides and isoprenes, as well as polyketide-nonribosomal peptide hybrids. A selection of secondary metabolites with pharmaceutical relevance is provided in Table 1.

Traditionally, bioactive secondary metabolites were discovered by screening single organisms or complex samples. For example, the immunosuppressive cyclosporine A was discovered 45 years ago from a Norwegian soil sample [11]. Today, genome mining studies have revealed a potentially large number of unknown secondary metabolite genes that are often not expressed under standard laboratory conditions. Thus far, activation of “silent” gene clusters has been achieved by overexpression of cluster-encoded or global regulators, epigenetic modification, and cocultivation experiments [reviewed in 9]. For example, cocultivation of *A. nidulans* with an actinomycete triggered production of orsellinic acid, and induced expression of the *A. nidulans* transcriptional regulator AfoA triggered production of the polyketide asperfuranone [12], [13]. These novel approaches will be of great value for identifying and engineering new secondary metabolites for biotechnological and pharmaceutical applications.

## Horizontal Gene Transfer Contributes to the Diversity of Fungal Metabolism

**Figure 1:**
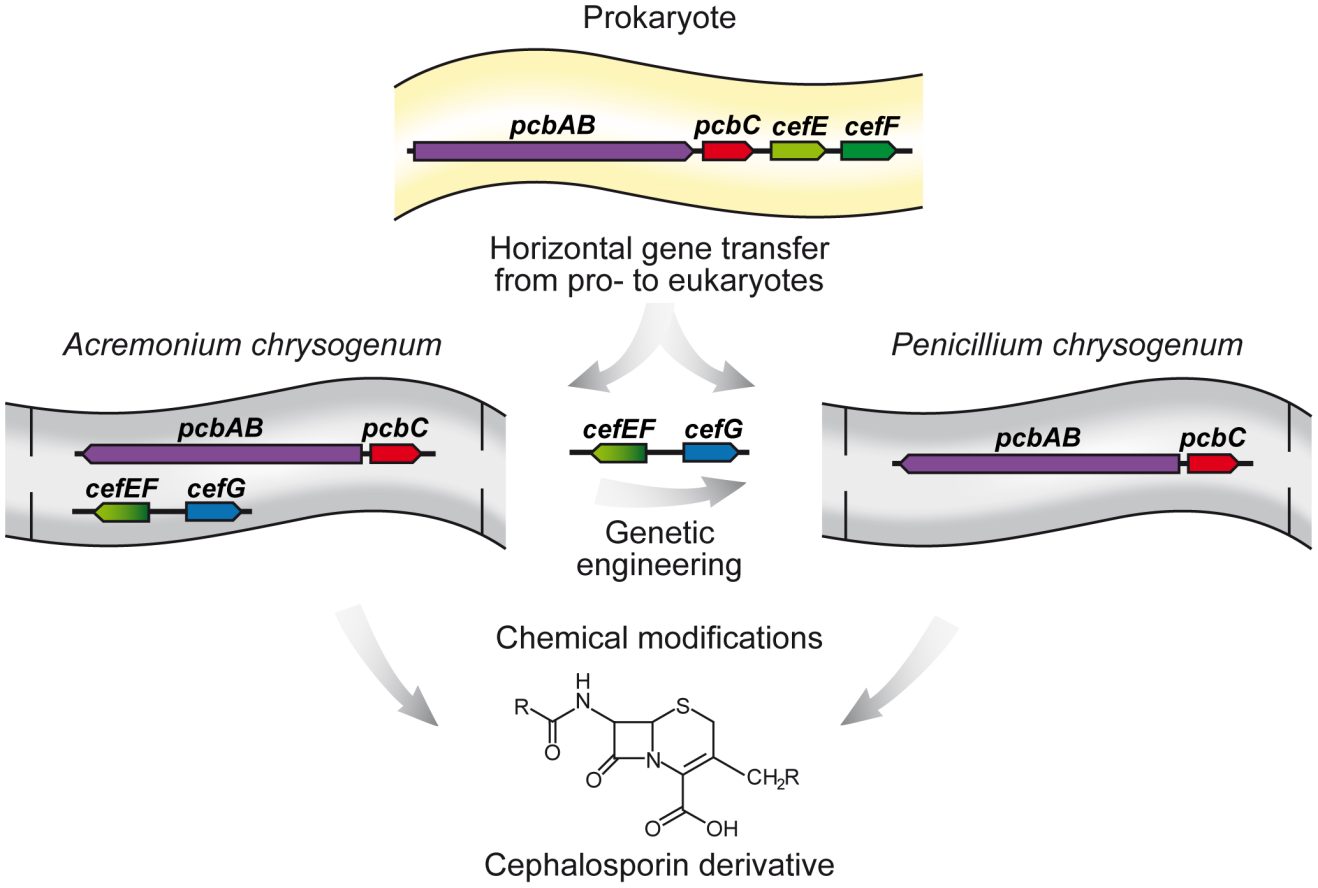
Horizontal gene transfer of beta-lactam biosynthesis genes from prokaryotes to eukaryotes. The antibiotic biosynthesis genes are derived either from gram-positive (e.g. *Streptomyces* spp.) or gram-negative (e.g. *Lysobacter* spp.) bacteria. The first two steps of both penicillin and cephalosporin C biosynthesis are catalyzed by the gene products of *pcbAB* and *pcbC. P. chrysogenum* harbors one additional gene, *penDE* (not shown), to perform the last step of penicillin biosynthesis, whereas *Ac. chrysogenum* has obtained several additional genes for production of cephalosporin C. Genetic engineering approaches have been used to introduce these genes into *P. chrysogenum*. Chemical engineering approaches have enabled the precursors of both the *Ac. chrysogenum* and *P. chrysogenum* biosynthesis pathway to be used for the production of new cephalosporin derivatives.

The immense diversity of secondary metabolites found in fungal species raises the intriguing question of their evolutionary origin. Lateral or horizontal gene transfer (HGT) across species barriers has long been known for bacteria but was thought to play a minor role in the evolution of eukaryotic species. However, recent data from genome sequencing studies have indicated that HGT has contributed considerably to this richness in metabolites [14], [15]. Investigation of 60 completely sequenced fungal genomes using strict phylogenomic criteria showed that 713 bacterial genes had been transferred to fungal genomes [16]. Several of these genes are involved in carbohydrate metabolism, allowing fungi to propagate under extreme environmental conditions. Early evidence that fungi acquired bacterial genes came from sequence comparison of beta-lactam antibiotic biosynthesis genes. The first two steps of beta-lactam antibiotic biosynthesis are catalyzed by a nonribosomal peptide synthetase and isopenicillin N synthase, leading to the intermediate isopenicillin N, which is common to all penicillins and their derivatives. Sequencing of the corresponding genes (*pcbAB* and *pcbC*) has provided evidence that these two genes are clustered in both bacteria and fungi (Figure 1). Furthermore, the clustered organization and the lack of any intronic sequences led to the assumption that bacterial beta-lactam antibiotic genes were transferred to fungal species such as *A. nidulans*, *Penicillium chrysogenum*, and *Acremonium chrysogenum*. The transfer of bacterial genes to a eukaryotic nucleus has a wide range of consequences. For example, the foreign genes have to adapt to the eukaryotic host system and its expression machinery. In prokaryotes, pathway-specific regulators commonly determine gene expression, while in eukaryotes, expression of secondary biosynthesis genes is often governed by global regulators. Good examples of such eukaryotic global regulators are LaeA and VeA, both part of a multi-subunit protein complex that controls the expression of genes, including those that encode beta-lactams, that are involved in fungal secondary metabolite processes [17], [18].

## Fungal Beta-lactam Antibiotics as a Key for Current and Future Treatment of Patients

Although fungi have most probably obtained genes for beta-lactam antibiotic biosynthesis through HGT from prokaryotes (Figure 1), they are more efficient producers of these secondary metabolites and, therefore, are preferentially chosen for industrial manufacturing. Currently, beta-lactam antibiotics are the major anti-infective agents worldwide, having an estimated world market of about 22 billion US dollars at the dosage form level [19]. In contrast to penicillin, which is mainly active against gram-positive bacteria, cephalosporin C is a broad-spectrum antibiotic that affects both gram-positive and gram-negative bacteria. Both antibiotics are produced by filamentous fungi, namely *P. chrysogenum* and *Ac. chrysogenum.* Both fungi share the first two steps of beta-lactam biosynthesis that are catalyzed by the gene products of *pcbAB* and *pcbC*, while the subsequent steps, resulting in either penicillin or cephalosporin C, are different. In *P. chrysogenum*, a single biosynthesis step is necessary to transform isopenicillin N into penicillin, and this is catalyzed by the *penDE* gene product. In contrast, several additional steps are needed in *Ac. chrysogenum* to generate cephalosporin C. The corresponding genes are part of a so-called “early” gene cluster, including *pcbAB* and *pcbC*, and a “late” gene cluster, comprising the genes *cefEF* and *cefG* [reviewed in 20]–[22]. Using genetic engineering approaches, genes from *Ac. chrysogenum* were transferred into *P. chrysogenum*, making this fungus an alternative producer of intermediates of cephalosporin C biosynthesis (Figure 1) [22].

Cephalosporin C as a natural product exerts only weak antibiotic activity. This activity has been gradually increased through the generation of semisynthetic derivatives, representing a good example of effective chemical modification of natural products [21]. Pharmaceutical companies have recently announced the development of cephalosporins that are particularly suitable for treating methicillin-resistant *Staphylococcus aureus* (MRSA), an increasing problem in hospitals. Recently, ceftobiprole became the first broad-spectrum cephalosporin with activity against MRSA to be assessed in late-stage clinical trials [23]. Furthermore, another cephalosporin derivative, ceftaroline, is one of only a few new antibiotics that have been recently launched [24], [25], indicating that cephalosporin derivatives remain extremely valuable antibiotics for current and future medical applications.

In summary, fungi can play a Janus-like role, being at the same time “friend” and “foe” to humans. In other words, they can not only act as serious pathogens themselves but can also produce a cornucopia of highly beneficial drugs and antibiotics. This unique ability of such fascinating filamentous fungal species thereby makes them of key interest in further biotechnological and pharmaceutical research.
